# Elimination of Onchocerciasis from Mexico

**DOI:** 10.1371/journal.pntd.0003922

**Published:** 2015-07-10

**Authors:** Mario A. Rodríguez-Pérez, Nadia A. Fernández-Santos, María E. Orozco-Algarra, José A. Rodríguez-Atanacio, Alfredo Domínguez-Vázquez, Kristel B. Rodríguez-Morales, Olga Real-Najarro, Francisco G. Prado-Velasco, Eddie W. Cupp, Frank O. Richards, Hassan K. Hassan, Jesús F. González-Roldán, Pablo A. Kuri-Morales, Thomas R. Unnasch

**Affiliations:** 1 Centro de Biotecnología Genómica, Instituto Politécnico Nacional, Reynosa, Tamaulipas, México; 2 Dirección General de Programas Preventivos, Centro Nacional de Programas Preventivos y Control de Enfermedades, Secretaría de Salud, Colonia Escandón, Delegación Miguel Hidalgo, Mexico City, México; 3 Onchocerciasis Elimination Program for the Americas, Guatemala City, Guatemala; 4 Consejería de Educación, Madrid, España, Leganés, Madrid, Spain; 5 Programa de Eliminación de la Oncocercosis en Chiapas, Departamento de Prevención y Control de Enfermedades Transmitidas por Vector, Secretaría de Salud Unidad Administrativa, Tuxtla Gutiérrez, Chiapas, México; 6 Department of Entomology and Plant Pathology, Auburn University, Auburn, Alabama, United States of America; 7 River Blindness Program, Carter Center, Atlanta, Georgia, United States of America; 8 Global Health Infectious Disease Research Program, Department of Global Health,University of South Florida, Tampa, Florida, United States of America; 9 Subsecretaría de Prevención y Promoción de la Salud, Secretaría de Salud, Colonia Juárez, Delegación Cuauhtémoc, México, D.F., México; McGill University, CANADA

## Abstract

**Background:**

Mexico is one of the six countries formerly endemic for onchocerciasis in Latin America. Transmission has been interrupted in the three endemic foci of that country and mass drug distribution has ceased. Three years after mass drug distribution ended, post-treatment surveillance (PTS) surveys were undertaken which employed entomological indicators to check for transmission recrudescence.

**Methodology/Principal findings:**

In-depth entomologic assessments were performed in 18 communities in the three endemic foci of Mexico. None of the 108,212 *Simulium ochraceum* s.l. collected from the three foci were found to contain parasite DNA when tested by polymerase chain reaction-enzyme-linked immunosorbent assay (PCR-ELISA), resulting in a maximum upper bound of the 95% confidence interval (95%-ULCI) of the infective rate in the vectors of 0.035/2,000 flies examined. This is an order of magnitude below the threshold of a 95%-ULCI of less than one infective fly per 2,000 flies tested, the current entomological criterion for interruption of transmission developed by the international community. The point estimate of seasonal transmission potential (STP) was zero, and the upper bound of the 95% confidence interval for the STP ranged from 1.2 to 1.7 L_3_/person/season in the different foci. This value is below all previous estimates for the minimum transmission potential required to maintain the parasite population.

**Conclusions/Significance:**

The results from the in-depth entomological post treatment surveillance surveys strongly suggest that transmission has not resumed in the three foci of Mexico during the three years since the last distribution of ivermectin occurred; it was concluded that transmission remains undetectable without intervention, and *Onchocerca volvulus* has been eliminated from Mexico.

## Author Summary

Onchocerciasis, or river blindness, is one of the neglected tropical diseases targeted by the international community for elimination. In Mexico, onchocerciasis was historically endemic in three foci, which included Northern Chiapias, Southern Chiapas and Oaxaca. Both the criteria for verification of elimination and for post-treatment surveillance developed by the international community rely heavily on the use of entomological metrics. The absence of evidence of ongoing transmission of the parasite three years after mass drug distribution has been halted is considered to be evidence that elimination efforts have been successful. In the present study, we report entomological assessments carried out in the three endemic foci in Mexico that were performed three years following the end of mass drug distribution in each focus. None of the over 100,000 *Simulium ochraceum* s.l. vector black flies collected from sentinel and extra-sentinel communities in these foci were found to contain parasite DNA, suggesting vector parasite contact was non-existent. This data suggest that elimination of onchocerciasis from Mexico has been achieved.

## Introduction

Onchocerciasis (river blindness) is caused by chronic infection with *Onchocerca volvulus*, a filarial nematode that is transmitted by *Simulium* spp. (Diptera: Simuliidae). The disease historically has constituted a serious public health concern and an enormous source of socio-economic disruption in many developing countries, most severely in sub-Saharan Africa and to a lesser extent in Latin America, where the parasite was introduced from Africa several centuries ago [1–6]. The current strategy for the elimination of onchocerciasis relies on mass treatment of endemic communities with ivermectin (Mectizan, donated by Merck & Co.). A variety of treatment regimens, i.e., quarterly and semi-annual treatment, have proven effective in interrupting transmission and eliminating the parasite throughout much of Latin America; semi-annual and annual treatments have also succeeded in isolated foci in Africa [7–12]. High coverage (≥ 85% of eligible persons), community-wide treatment of residents is believed to be sufficient to reduce the load of microfilariae in human hosts below the threshold that can sustain transmission by black fly vectors, thus locally eliminating the infection [7].

The elimination guidelines set forth by the Onchocerciasis Elimination Program for the Americas (OEPA) and the World Health Organization (WHO) use the prevalence of *O*. *volvulus* infective stage larvae (L_3_) in the black fly vectors as a major metric for determining whether or not transmission has been successfully interrupted in an endemic community [13, 14]. In Latin America, the threshold used for declaring interruption of transmission is an upper bound of the 95% confidence interval (95%-ULCI) for the point estimate of the prevalence of vectors carrying L_3_ of less than 1/2,000 per endemic community [14]. At least 6,000 flies must be tested and all must be found to be L_3_-free to satisfy this standard [15,16]. In addition to the 1/2,000 infective fly threshold, OEPA recommends the use of the Annual Transmission Potential (which in the present situation is equivalent to seasonal transmission potential [STP]) to assess the status of *O*. *volvulus* transmission, because transmission potentials take into account both the biting rate and the prevalence of infective flies. OEPA/WHO verification guidelines for onchocerciasis elimination stipulate that in areas where transmission has been interrupted and mass drug distribution has been stopped, a post-treatment surveillance (PTS) period of at least 3 years is needed [14, 17]. If surveys conducted after the PTS period show no evidence for recrudescence of transmission, then *O*. *volvulus* is considered to have been eliminated, and the resident population is no longer at risk.

In Mexico, onchocerciasis was historically endemic in three distinct foci; Southern Chiapas, Northern Chiapas, and Oaxaca (Table 1). In 1960, a total of 20,090 individuals harbored nodules (a prevalence of 15%); 135 individuals blinded by onchocerciasis were reported (representing a prevalence of 0.1%). In the Oaxaca focus 5,800 cases (i.e. individuals diagnosed positive for onchocerciasis by any of the available methods, Mazzotti reaction, nodule palpation, or skin biopsies (snips) during active case finding campaigns conducted by the Mexican onchocerciasis brigades) were reported in 1960. This represented a prevalence of 13% in the at risk population of 45,000 individuals residing in 154 communities. In contrast, in 1960, the Northern Chiapas focus had just 4,000 imported cases (residents that had regularly visited other foci where they likely acquired the infection) in an at risk population of 22,500 individuals residing in 133 communities, representing a prevalence of 18%; by 1993 only 180 cases were reported in a population of 15,539 at risk individuals in this focus, representing a prevalence of just 1%. The Oaxaca and Northern Chiapas foci were therefore considered as hypo-endemic for onchocerciasis, as the prevalence in these foci was less than 20%.

**Table 1:** The epidemiological situation in the three onchocerciasis endemic foci in Mexico.

Focus/Year of evaluation	No. of “new” clinical cases*	No. of individuals at risk	Prevalence of Infective flies/2,000 ^¶^	Seasonal transmission potential ^¶^
Northern Chiapas^1^/ 1993	13	15,539	ND	ND
Northern Chiapas^1^/ 1999–2001	0	21,572	0.4 (0.0–0.90)	1.0 (0.0–2.2)
Northern Chiapas^1^/ 2005	0	7,092	0 (0.09)	0 (0.05)
Northern Chiapas^#^/ 2010	0	7,125^&^	0 (0.3)	0 (4.4)
Southern Chiapas^2^/ 1993–1991	904	190,744	1.8 (0.9–3.3)^&^	95.2^&^
Southern Chiapas^2^/ 1999–2001	274	219,923	0.4 (0.2–0.90)	1.2 (0.6–2.8)
Southern Chiapas^2^/ 2011	9	114,024	0 (0.06)	0 (1.0)
Southern Chiapas^#^/ 2014	0	117,825^&^	0 (0.1)	0 (1.7)
Oaxaca^3^/ 1993	316	64,426	ND	ND
Oaxaca^3^/ 1999–2001	1	65,447	0.7 (0.4–1.2)	3.2 (1.9–5.8)
Oaxaca^3^/ 2008	0	44,919	0 (0.07)	0 (1.9)
Oaxaca^#^/ 2011	0	44,919^&^	0 (0.1)	0 (1.2)

Geographical extension (2000): 1) 1,172.10 km^2^; 2) 13,901.3 km^2^; 3) 4,250,0 km^2^.

* “New” clinical onchocerciasis cases were defined as those individuals diagnosed positive by Mazzotti reaction, nodules, or skin biopsies (‘snips’) for the first time.

^¶^ The upper value represents point estimate and the lower value in parentheses represents the 95%-confidence interval. When the point estimate was 0 only the upper limit of confidence interval is presented.

^&^ Las Golondrinas [29].

^#^ The present study.

^&^ Population no longer at risk of infection.

The Southern Chiapas focus was the major focus in Mexico, given its large size (12,000 km^2^) and a well-documented history of intense transmission (Table 1). In 1960, 26,003 cases were reported in an at risk population of 61,619 individuals residing in 837 communities were reported (a prevalence of 42%). In 1999, 22,361 cases were reported, of which 274 were classified as “new” clinical cases (i.e. individuals diagnosed positive by Mazzotti reaction, nodules, or skin biopsies for the first time, during active case finding campaigns conducted by the onchocerciasis brigades), while 782 individuals harbored nodules in an at risk population of 219,923; 31 individuals blinded by onchocerciasis were reported [18–20]. The prevalence of cases, “new” clinical cases, nodules, and onchocercal blindness were 10%, 0.1%, 0.3%, and 0.01%, respectively. In 2012, Mexico was surpassed only by Guatemala in the Americas in terms of the at risk population for onchocerciasis. The total population at risk in Guatemala and Mexico together represented 71% of Latin America´s total at risk population of 565,232 individuals.

Onchocerciasis was discovered in the Americas in 1915 by Dr. Rodolfo Robles, who described the first clinical cases in Guatemala. In Mexico, the first cases of onchocerciasis were documented in Southern Chiapas in 1923. The disease was probably introduced to this area due to the seasonal migration of coffee workers from the endemic foci of Guatemala. Similarly, the Oaxaca and Northern Chiapas foci may have resulted from the expansion of coffee cultivation into these areas and the corresponding migration of workers from the established foci of Southern Chiapas and Guatemala [21]. One of the first programs to combat onchocerciasis in the world was established in Mexico in 1930; this program has been operating continuously since then. From 1930 through 1946, the Mexican onchocerciasis control program carried out sporadic vector control campaigns, treating breeding sites with creosote as a larvicide to reduce vector populations, and nodulectomy campaigns (removal of nodules harboring adult worms) to reduce the most severe cases of the disease. Administration of diethylcarbamazine (DEC) began in 1947, when it was tested in six infected individuals. In 1949, DEC began to be provided to all clinical cases of onchocerciasis. This was augmented in 1952 with sporadic applications of dichlorodiphenyltrichloroethane (DDT) to control the vector population [21]. In 1990, ivermectin (Mectizan, Merck & Co., Inc., Whitehouse Station, NJ) replaced DEC. The Mexican program initially used ivermectin only in symptomatic individuals. However, beginning in 1997, ivermectin was provided to all individuals living in endemic communities, using a strategy of administering two rounds of treatments per year (semi-annual regimen); this was followed by a distribution of four rounds of treatment per year (quarterly regimen) from 2003 through 2011 in the Southern Chiapas focus [18]. The increase in the frequency of ivermectin treatments proved to be a good strategy, accelerating the interruption of parasite transmission in this focus [8]. Recent in-depth epidemiological studies based on entomological, parasitological, serological, and ophthalmological surveys conducted in individuals of endemic communities have demonstrated the interruption of parasite transmission in all three endemic foci in Mexico [18–20]. These results led to the cessation of the treatment with ivermectin by the Ministry of Health of Mexico. The endemic communities then entered the post-treatment surveillance (PTS) period.

Here, we present the results of PTS entomological surveys carried out in the three endemic foci of Mexico. Taken together, the results demonstrate that transmission has not resumed in the three years since the last distribution of ivermectin occurred. Mexico has entered the post-endemic era and now appears to be free of the scourge of onchocerciasis.

## Materials and Methods

### Study sites

**Fig 1:**
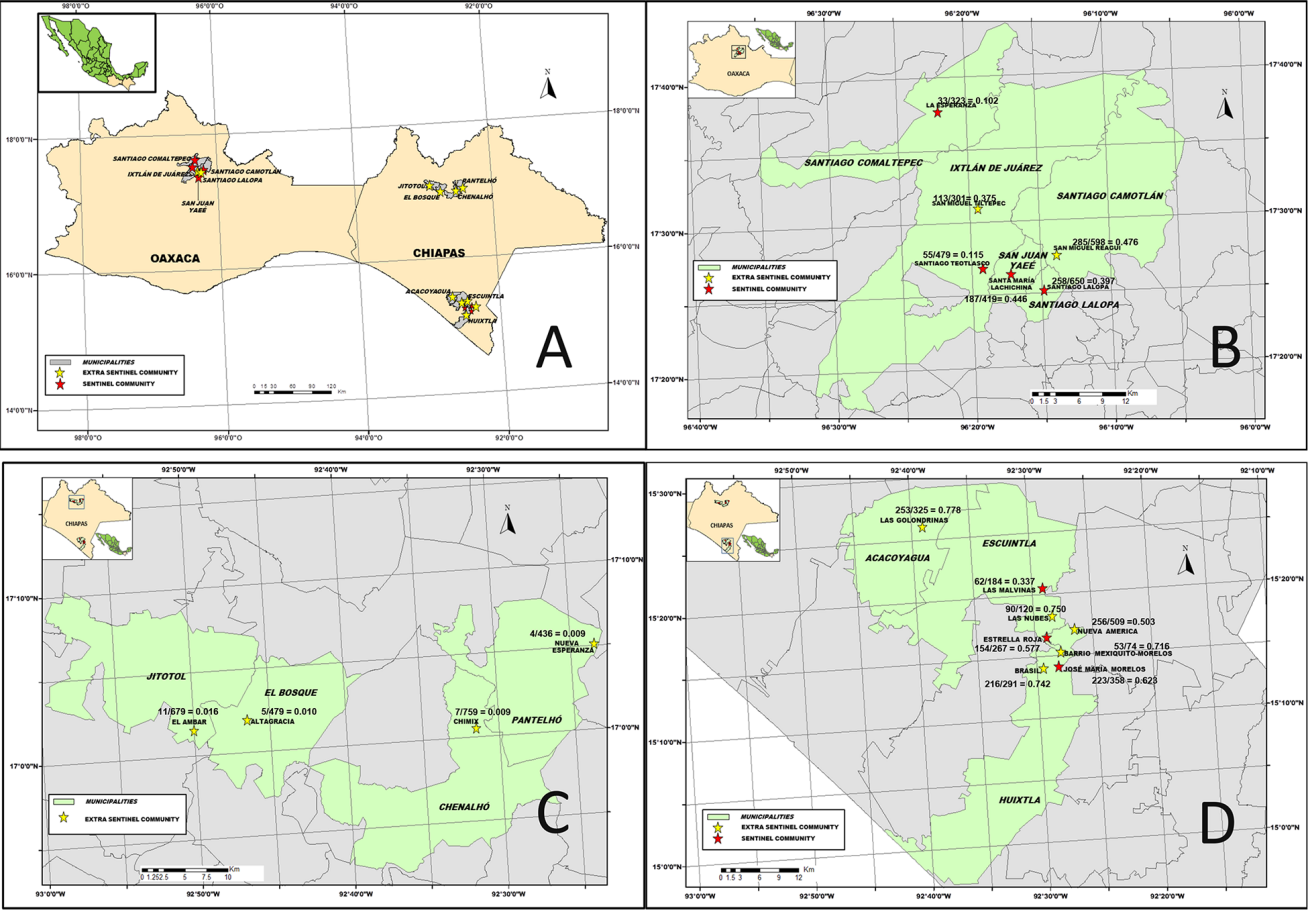
Location, number of cases, and population at risk when ivermectin distribution began at the study sites: Panel A: Map of the Southern Mexico states showing the three endemic foci for onchocerciasis. Panels B-D: the eighteen sentinel and extra-sentinel communities in Oaxaca (Panel B; prevalence data from 1995), Northern Chiapas (Panel C; prevalence data from 1999), and Southern Chiapas (Panel D; prevalence data from 1995).

**Fig 2:**
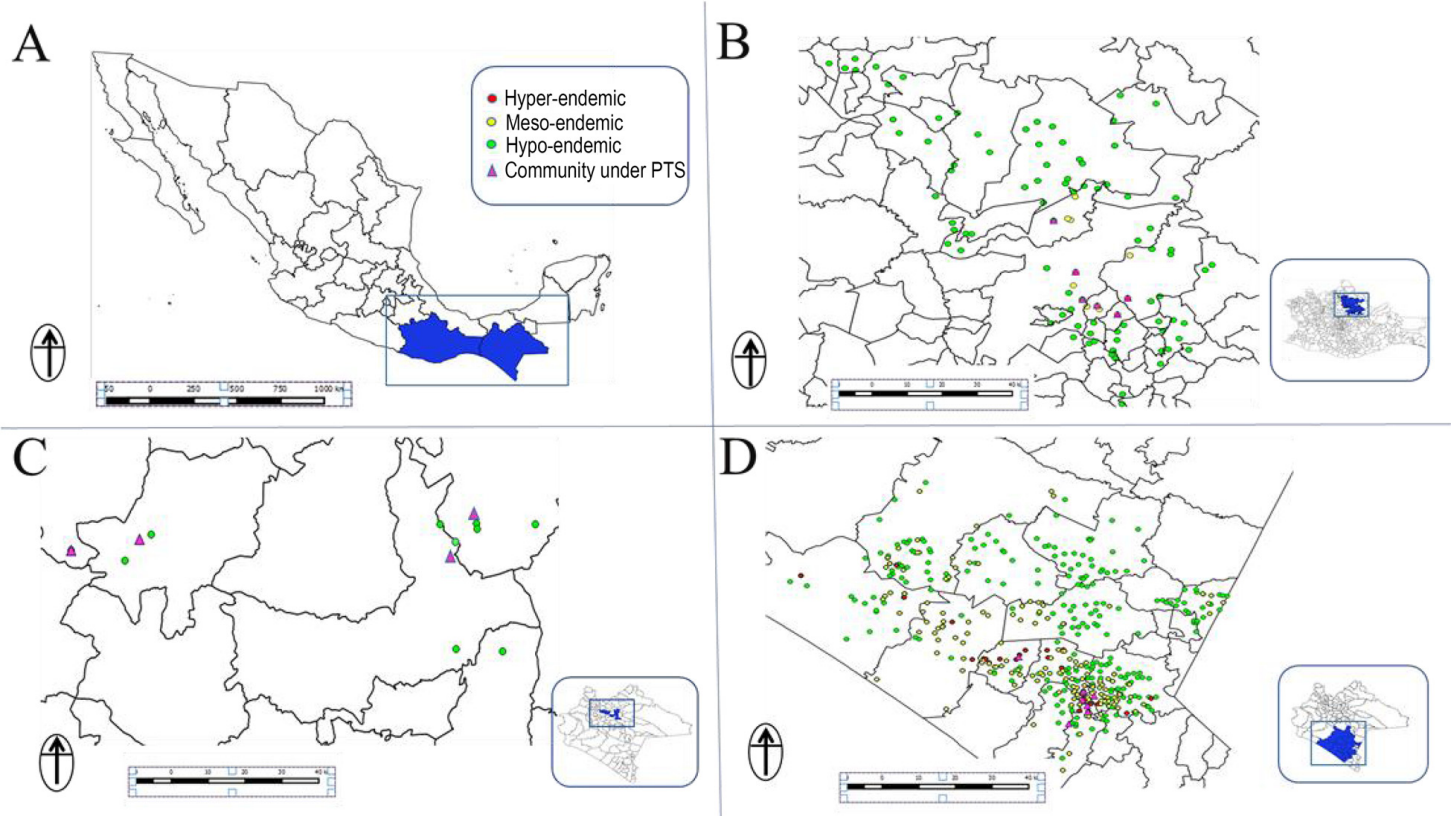
The onchocerciasis communities under post-treatment surveillance (PTS) phase in Mexico: Panel A: Map of the Southern Mexico states showing the two endemic States for onchocerciasis. A total of 98 communities were under PTS phase in the Oaxaca focus (Panel B). In addition, 13 and 559 communities were also under PTS phase in the Northern (Panel C) and Southern Chiapas foci, respectively (Panel D).

Flies were collected using human attractants from 18 sentinel and extra-sentinel communities as previously described [7, 8]. Four out of 13 endemic communities in the Northern Chiapas focus, 6 out of 98 endemic communities in the Oaxaca focus, and 8 out of 559 in the Southern Chiapas focus were included in the surveys (Fig 1). All communities were previously either meso- or hyper-endemic for onchocerciasis, and they were generally the communities with the most intense transmission in each focus before interventions began (Fig 2). In the two endemic states of Mexico, 39 communities were hyper-endemic and 220 and 411 were meso and hypo-endemic respectively. Meso-endemic communities were defined as having a historical onchocerciasis prevalence of more than 20% but less than 60% while hyper-endemic communities had a historical prevalence of 60% or higher. Hypo-endemic communities were those with a historical prevalence less than or equal to 20%.

**Fig 3:**
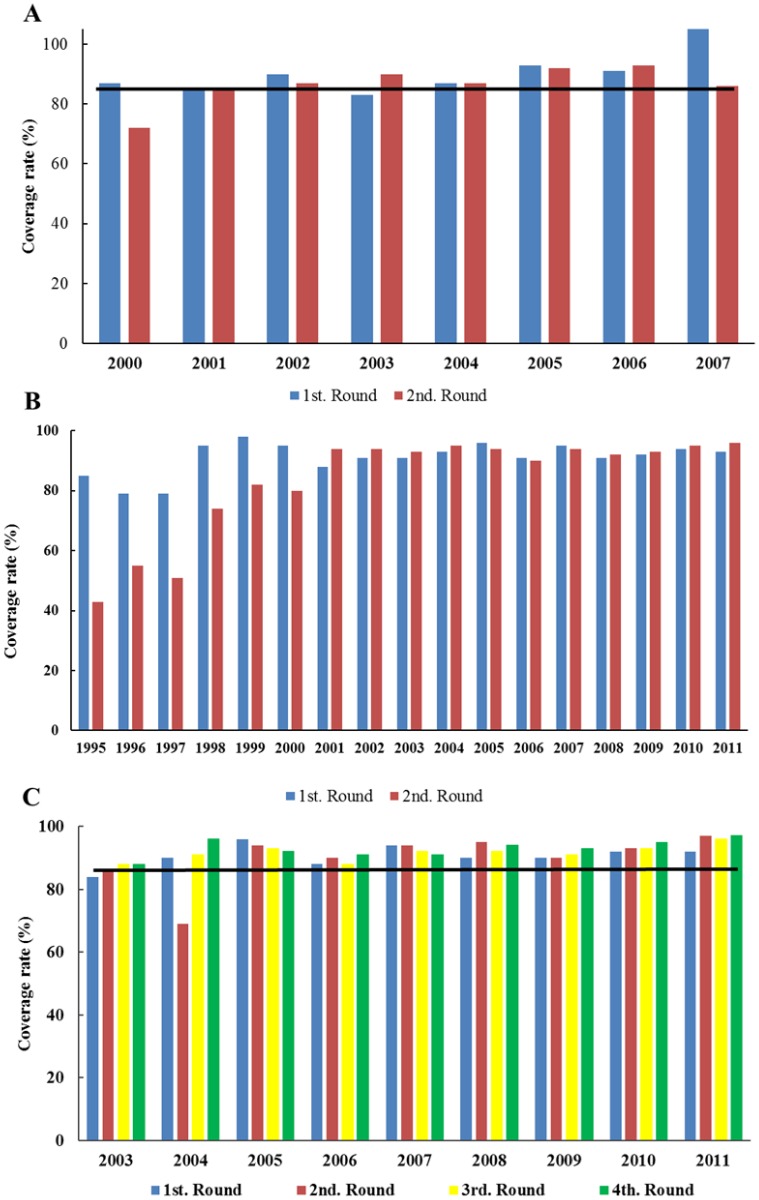
Mass drug administration (MDA) with ivermectin in two endemic foci: Coverage rate, expressed in percent, of the eligible population. The eligible population excluded pregnant and lactating women and children under 5 years of age. These groups represented 9.5% of the total population during the last year of MDA in the Southern Chiapas focus in 2011. Panel A: Semi-annual treatment regimen was employed in Northern Chiapas from 2000 through 2007 when MDA ceased. Panels B-C. Semi-annual regimen was employed in Southern Chiapas from 1995 through 2011 (Panel B; Figure taken from Rodriguez-Perez et al., 2013 [18]); in addition, quarterly treatment was employed in 50 communities from 2003 through 2008 and in 163 communities from 2009 through 2011 when MDA ceased (Panel C). The semi-annual treatment regimen was employed in Oaxaca from 1996 through 2008 when MDA ceased (see Fig 2 in Rodriguez-Perez et al., 2010 [20]). The line at 85% indicates the coverage needed to be maintained in order to interrupt transmission.

In the three endemic foci, the ivermectin treatment regimen was generally provided on a semi-annual basis. A quarterly treatment regimen was employed from 2003–2011 in communities of the Southern Chiapas focus, as described above. MDA coverage rates (percent) of the eligible population in the three foci are summarized in Fig 3.

### Ethics statement

All procedures involving use of humans for fly collections were reviewed and approved by the Bioethics Committee of the Center for Research and Development in Health Sciences of the Autonomous University of Nuevo León (Monterrey, Nuevo León, Mexico). Written informed consent was obtained from all fly collectors.

### Entomologic surveys

Black fly collection was carried out by two teams in each community, with each team consisting of a fly collector and a human attractant. One team was located at a randomly selected location within the community while the second team conducted collections at nearby coffee plantation. Black fly collections were performed during the dry seasons of November 2009 through February 2010, January through March 2011, and December 2013 through May 2014 in the foci of Northern Chiapas, Oaxaca, and Southern Chiapas, respectively. These collection periods coincided with the peak *Simulium ochraceum* sensu lato population densities and the peak *O*. *volvulus* transmission season [22]. *S*. *ochraceum* s.l. is the major vector of *O*. *volvulus* in Mexico. No other species of black fly has been documented to be an important vector in the Mexican foci [22].

Collections were performed during the first 50 min of each hour, beginning at 11:00 h and ending at 16:50 hrs. Collectors received ivermectin 1 week before beginning the collection process. The black fly collections were supervised by federal health officials to ensure that collections were conducted throughout the entire 50 min collection period of each hour; the remaining 10 min of each hour was utilized as a break period. The officials also ensured that the black flies were stored appropriately. The black flies were transported every hour to a field station for observation under a dissecting microscope. The flies were identified to the species level; flies other than *S*. *ochraceum* s.l. and any fly containing evidence of having taken a recent blood meal were discarded. The number of *S*. *ochraceum* s.l. collected by each team in each 50-minute period was recorded. The *S*. *ochraceum* s.l. females were then combined into pools using a pool size of 50 flies/pool for the samples collected in the Oaxaca and Northern Chiapas foci and a pool size of 200 flies/pool for those samples collected in the Southern Chiapas focus.

### PCR pool-screening assay

Black flies were collected before they began blood-feeding. The landing rate as measured from the collections was taken as an estimate of the biting rate, although this probably overestimated the biting rate, as a proportion of the landing flies in a natural setting do not successfully obtain a blood meal. Flies were combined into pools as described above and the heads and bodies separated by freezing and agitation, as previously described [7, 8, 23, 24]. The separated bodies were tested for *O*. *volvulus* parasites by using a PCR assay specific for *O*. *volvulus*, as previously described [7, 8, 23, 24]. Screenings initially focused on pools of bodies, as previous studies have shown that infection rates in bodies, which contain multiple life cycle stages of the parasite, provide a more sensitive indicator of parasite-vector contact than testing heads, which only contain L_3_ larvae [23, 25]. PoolScreen v2.0 was used to estimate the upper bound of the 95% confidence interval for the prevalence of flies carrying *O*. *volvulus* [26]. The seasonal transmission potential (STP) was calculated as the product of the seasonal biting rate, the proportion of flies carrying L_3_ and the average number of L_3_ larvae in each infective fly. After multiple rounds of ivermectin treatment, the number of L_3_ present in each infective fly was assumed to be one, as previously described [7, 8, 18]. The seasonal biting rate was calculated as the product of the arithmetic mean of the number of flies collected per person per day and the total number of days in the transmission season. Because *S*. *ochraceum s*.*l*. females were not collected throughout the year, it was not possible to precisely calculate the annual transmission potential (ATP). However, given the low abundance of vector black flies present outside the normal transmission season, the transmission potential outside of the peak transmission period is probably zero or near zero [22]. The STP (transmission occurring during the peak transmission dry season of December through May) thus likely represented a fairly accurate estimate of the ATP.

## Results

In the Northern Chiapas focus, totals of 5,731 and 5,476 host-seeking *S*. *ochraceum* s.l. females were collected from four extra-sentinel communities in the community and coffee plantation collection sites respectively. These were divided into a total of 230 pools, each containing a maximum of 50 individuals for PCR analysis. In the Oaxaca focus, a total of 11,148 and 17,494 host-seeking *S*. *ochraceum* s.l. females were collected in the community and coffee plantation sites, respectively. These were divided into a total of 582 pools, each containing a maximum of 50 individuals for PCR. Finally, a total of 40,001 and 28,362 host-seeking *S*. *ochraceum* s.l. females were collected in the community and coffee plantation sites of Southern Chiapas respectively. These were divided into a total of 362 pools, each containing a maximum of 200 individuals. The number of vectors collected was sufficient to comply with the WHO/OEPA guideline of having at least 6,000 flies tested from each focus.

None of the pools of *S*. *ochraceum* s.l. collected in 2010 and 2011 in the Northern Chiapas and Oaxaca foci (11,207 and 28,642 flies respectively) were found to be positive in the PCR assay. Thus, the associated upper limit of the 95% confidence interval (95% ULCI) for the prevalence of flies carrying *O*. *volvulus* were 0.3 and 0.13/2,000 flies for Northern Chiapas and Oaxaca respectively, both of which were below the threshold of a 95%-ULCI of 1/2,000 mandated by the international community as sufficient to declare that transmission had been eliminated (Table 2). Similarly, the 68,383 flies collected from the Southern Chiapas focus were also all found to be negative for parasite DNA. In this case, the 95%-ULCI for the prevalence of infection in the vector was just 0.1/2000 (Table 2).

**Table 2:** Entomological parameters in the three foci of onchocerciasis in Mexico.

Focus	Collection sites*	*Simulium ochraceum* s.l. collected	Pools examined	PCR positive pools	Seasonal biting rate ^¶^	Prevalence of Infective flies/2,000^¶^	Seasonal transmission potential^¶^
Northern Chiapas	4	11,207	230^§^	0	8,732 (8,064–9,446)	0 (0–0.30)	0 (0–1.3)
Oaxaca	6	28,642	582^§^	0	18,218 (15,668–21,155)	0 (0–0.13)	0 (0–1.2)
Southern Chiapas	8	68,363	362^&^	0	33,992 (32,050–36,044)	0 (0–0.10)	0 (0–1.7)

* Northern Chiapas: El Ambar, Alta Gracia, Chimix, and Nueva Esperanza; Oaxaca: Santiago Teotlaxco, Tiltepec, San Miguel Reagui, Santiago Lalopa, La Chichina, and La Esperanza Comaltepec; Southern Chiapas: Brasil, Mexiquito, Jose Maria Morelos, Estrella Roja, Ampliación Malvinas, Las Golondrinas, Las Nubes II, and Nueva América.

^§^ Each pool contained a maximum of 50 flies.

^&^ Each pool contained a maximum of 200 flies.

^¶^ The upper value represents point estimate and the lower value in parentheses represents the 95%-confidence interval.

The point estimate for the STP in all foci was zero. The 95%-ULCI for the STP was 1.3 and 1.2 L_3_/person/season in the Northern Chiapas and Oaxaca foci respectively, while in the Southern Chiapas focus the 95%-ULCI for the STP was 1.7 L_3_/person/season (Table 2). These values were well below the ATP breakpoint for transmission, which has been estimated by various sources to be between 5–20 L_3_/person/year [27, 28]. Taken together, these data suggest that no parasite-vector contact was occurring in any of the foci in Mexico three years following the end of mass Mectizan distribution.

## Discussion

Onchocerciasis was historically endemic in three foci in Southeastern Mexico; Northern Chiapas, Southern Chiapas and Oaxaca. The smallest of these, Northern Chiapas, was also the first in which transmission of *O*. *volvulus* was reported to have been interrupted following exhaustive clinical, epidemiological and entomological surveys [19]. This occurred in 2007, following 10 years of semi-annual Mectizan mass treatment of the at-risk communities [19, 23, 29, 30]. Northern Chiapas was followed in 2008 by the second largest focus in Mexico, Oaxaca (after 13 years of semi-annual treatment) [7, 20], and finally by the largest focus, Southern Chiapas, in 2011 (following 17 years of semi-annual and quarterly treatments aimed at hastening onchocerciasis elimination) [18]. Following the declaration of the interruption of transmission in each of these foci, the Mexican Ministry of Health accepted the recommendations of the Program Coordinating Committee (PCC) of OEPA, and discontinued ivermectin treatments in these foci. Following the recommendations by OEPA and WHO [13, 14, 17], post treatment surveys were conducted three years after the end of mass drug distribution activities in each focus. These surveys focused on entomological monitoring for evidence of transmission, as this represents the earliest indicator of ongoing transmission available [14]. The data collected from these surveys, which are reported here, uncovered no evidence of transmission three years after treatment was halted in any of the three foci in Mexico. These results suggest that parasite transmission has not resumed in the three years since drug pressure was removed, and that *O*. *volvulus* has therefore been eliminated from each of the foci. Mexico as a country can now declare national elimination of onchocerciasis, and request WHO for verification of elimination.

The 95%-ULCI for the prevalence of flies carrying *O*. *volvulus* parasites in all three foci was found to be much less than the 1/2000 threshold developed by OEPA and WHO; furthermore more than 10,000 flies were tested from each focus, satisfying the criterion developed by WHO for verifying onchocerciasis elimination [17]. However, it has been pointed out that the risk of recrudescence is in part dependent upon the number of vectors biting residents of affected communities, and that measurements of ATP, which take biting rates into account, may be a better indicator of the risk of recrudescence than the prevalence of infection in the vector population alone [31]. Estimates of the ATP necessary to maintain the parasite population (the transmission breakpoint) range from 5 to 54 L_3_/person/year based on mathematical modeling [27] and from 7.6 to 18 L_3_/person/year based on field observations [28]. In the PTS surveys reported here, the point estimates for the STP for all foci were all zero, with the 95%-ULCI for all foci falling below 2 (Table 2). Thus, these data confirm that three years following the cessation of mass drug treatments in the population, transmission values remained well below the transmission breakpoint.

The Northern Chiapas focus historically had little autochthonous transmission; onchocerciasis cases in this focus were believed to have resulted from importation from Southern Chiapas and/or Guatemala. Oaxaca, while clearly having autochthonous transmission, was a much smaller focus than Southern Chiapas, both in terms of area and at risk population (Table 1). Interruption of transmission was successfully achieved in the two smaller foci using a semi-annual ivermectin treatment regimen. In contrast, progress in Southern Chiapas was delayed relative to Northern Chiapas and Oaxaca, and it was necessary to move to a quarterly treatment regimen to accelerate the process towards the interruption of transmission (Fig 3 Panel D). In all foci, the major challenge faced by the program was in obtaining and maintaining the coverage rates needed to ensure interruption of transmission. Mainly, this challenge resulted from two classes of individuals who were not receiving ivermectin; those who were chronically absent during the days their community was treated and those who were consistently non-compliant with respect to the program. A third untreated group were those ineligible for treatment, i.e. individuals under the age of five, or pregnant or lactating women. Individuals in the latter group generally received treatment once reaching eligible age, or when no longer pregnant or lactating. With respect to those who were chronically absent during the treatment period, coverage was found to improve significantly when the program moved from semi-annual to quarterly treatments. This change of strategy allowed the brigades to locate and treat people who were absent during the semi-annual visits. The brigades also performed many educational campaigns promoting health and preventing disease; these had the effect of informing the population reaching the people who were not compliant at the beginning of the program and convincing them of the importance of being treated, improving the coverage rate as a whole.

It is unlikely that onchocerciasis will be re-introduced to the Mexican foci from elsewhere, as these foci are well isolated from others in Latin America, and, unlike some species in Africa, the vectors responsible for transmission in Latin America do not migrate seasonally [32]. Similarly, migrant workers who cross the Mexico–Guatemala border are unlikely to pose a threat for re-introduction of the infection, as both nations have now interrupted transmission [33]. Furthermore, transmission has already been interrupted in most of the other foci in the Americas, which themselves are entering the post-treatment surveillance phase [33]. Nonetheless, it will be important to continue some surveillance activities for the next few years to ensure that transmission does not re-occur.

The studies reported above report collections that were carried out at a number of sentinel and extra-sentinel communities in each focus and do not represent comprehensive surveys of all communities at risk in each focus. For example, the total population of the 18 communities studied here was 6,738 individuals, which represents only 4% of a total at risk population in the 670 communities under PTS. Thus, although the communities chosen were generally those with the most intense transmission before the program began, and thus are expected to represent the worst case scenario, it is still possible that a low level of transmission still might occur in communities that were not included in the surveys. Indeed, mathematical models predict low levels of transmission are likely to occur, but that the reproductive rate will remain well below 1.0 and the overall parasite population would inevitably decline to extinction [14]. In light of the PTS entomologic findings in the three endemic foci, it appears that the parasite reproductive rate is now at a negligible level. This has resulted in a level of transmission that is no longer detectable, even in the absence of intervention, making it probable that the parasite population will not be able to recover even in the absence of any control measures. Despite this, the Mexican Ministry of Health has recognized it will be prudent to continue to conduct periodic clinical and entomological surveys in the formerly endemic states to ensure that transmission does not recrudesce [34]. The recent development of traps that can replace human landing collections for *S*. *ochraceum* s.l., the major vector of onchocerciasis in Mexico [35] should facilitate this process.

**Fig 4:**
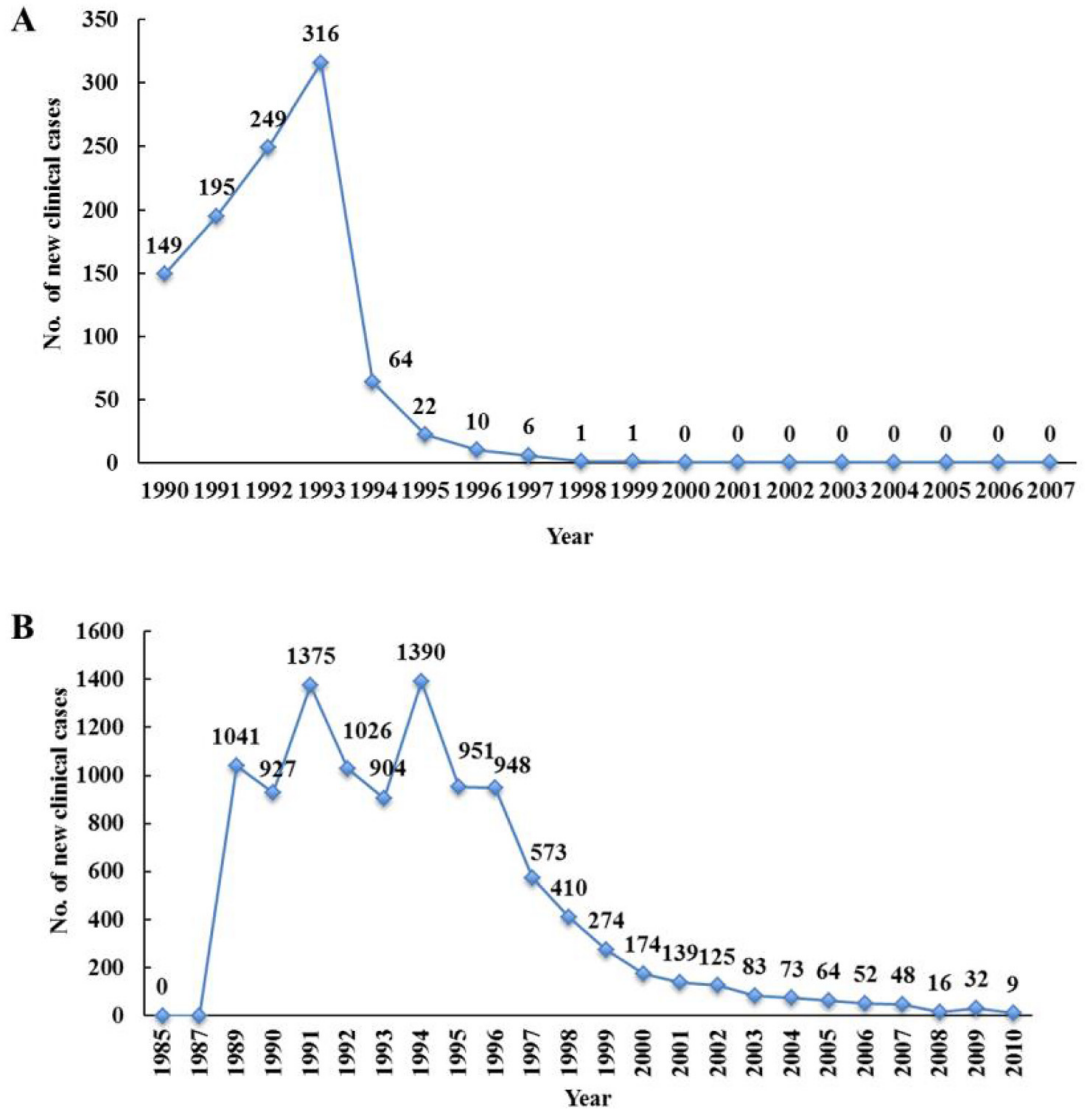
Number of new clinical cases in two endemic foci: The number of new clinical cases, (individuals diagnosed for the first time as positive by Mazzotti reaction, nodules, or skin biopsies) in the Oaxaca focus (Panel A) in the Southern Chiapas focus (Panel B; Figure taken from Rodriguez-Perez et al., 2013 [18]). The number of new clinical cases in the Northern Chiapas focus can be found in Fig 2 of Rodriguez-Perez et al., 2010 [19].

Additional data suggest that clinical onchocerciasis has also been eliminated in three endemic foci (Fig 4). There was a near absence of new clinically defined cases of onchocerciasis during the last years before the PTS phase began. Only five new clinical cases (i.e., individuals diagnosed positive for nodules or skin microfilariae for the first time) were reported in 1996 in the Northern Chiapas focus, while just nine new clinical cases were reported in Southern Chiapas in 2010 (Fig 4; Panel A). Similarly, no new clinical cases were reported in the Oaxaca focus from 2000 through 2007 (Fig 4; Panel B). These findings suggest that onchocerciasis no longer represents a health problem in the formerly endemic communities in Mexico.

In addition to Mexico, the programs of Ecuador [36] and Guatemala [37–39] have reported success in eliminating transmission of *Onchocerca volvulus* by the use of community-wide Mectizan distribution. The data presented here suggest that transmission interruption has been achieved in Mexico, resulting in elimination of this disease from the entire country. Coupled with recent studies in Mali, Nigeria, Senegal, Uganda and Northern Sudan that have indicated that ivermectin distribution may lead to focal elimination of onchocerciasis in certain African settings [40, 9–11, 41, 42], these findings give hope to the concept that worldwide elimination of this parasite is indeed possible.
